# Insulin resistance in people living with HIV is associated with exposure to thymidine analogues and/or didanosine and prior immunodeficiency

**DOI:** 10.1186/s12879-022-07485-1

**Published:** 2022-05-28

**Authors:** Julie Høgh, Malene Hove-Skovsgaard, Marco Gelpi, Anne Marie Reimer Jensen, Jan Gerstoft, Thomas Benfield, Heidi Storgaard, Susanne Dam Nielsen

**Affiliations:** 1 Department of Infectious Diseases, Copenhagen University Hospital-Rigshospitalet, Copenhagen, Denmark; 2 Department of Clinical Medicine, Faculty of Health and Medical Sciences, University of Copenhagen, Copenhagen, Denmark; 3 Department of Infectious Diseases, Copenhagen University Hospital-Amager and Hvidovre, Hvidovre, Denmark; 4 Steno Diabetes Center Copenhagen, University of Copenhagen, Herlev, Denmark

**Keywords:** HIV-infection, Diabetes, Insulin resistance, Comorbidity, Antiretroviral therapy

## Abstract

**Background:**

As people living with HIV (PLWH) are growing older, there is increased incidence of metabolic diseases, including type 2 diabetes mellitus, for which insulin resistance is a key determinant. In this study, we aimed to investigate risk factors associated with insulin resistance in PLWH.

**Methods:**

We included well-treated PLWH without hepatitis co-infection, and with available fasting serum insulin and plasma glucose (n = 643) from the Copenhagen Comorbidity in HIV Infection Study. Insulin resistance was calculated using the homeostasis model assessment of insulin resistance (HOMA-IR). We investigated the association between risk factors and high HOMA-IR in a logistic regression model adjusted for age, sex, abdominal obesity, smoking status, and origin. When including use of thymidine analogues and/or didanosine in the model, we also adjusted for time with HIV.

**Results:**

Median (IQR) age of PLWH was 52 years (46–61), and 87% (n = 557) were male. Median (IQR) HOMA-IR was 1.86 (1.23–3.14) mmol/L × mU/L. Risk factors significantly associated with high HOMA-IR included older age, BMI ≥ 25, abdominal obesity, waist circumference, use of thymidine analogues and/or didanosine, time with HIV, and CD4^+^ nadir < 200 cells/µL.

**Conclusions:**

Insulin resistance in PLWH is associated with both use of thymidine analogues and/or didanosine and prior immunodeficiency suggesting that increased attention on blood glucose in these patients could be beneficial.

## Background

The population of people living with HIV (PLWH) is growing older [1], and, accordingly, there is an increasing incidence of age-related non-AIDS comorbidities including diabetes mellitus type 2 (T2DM) [2–5]. Insulin resistance is a key determinant of T2DM and has previously been associated with age, overweight, and obesity [6]. Especially, abdominal obesity is thought to be associated with a high risk of developing insulin resistance [7, 8]. In PLWH, exposure to older generation antiretroviral therapy (ART) has been associated with both the development of T2DM and long-lasting alterations of body fat from subcutaneous adipose tissue to visceral adipose tissue [9–12].

In this study, we determined insulin resistance in PLWH and aimed to investigate traditional and HIV-specific risk factors associated with insulin resistance. Additionally, we investigated the potential effect modification of prior exposure to older generation ART defined as previous use of thymidine analogues and/or didanosine.

## Methods

### Study population and demographics

The Copenhagen Comorbidity in HIV Infection (COCOMO) Study is a non-interventional cohort study that included PLWH from the greater Copenhagen area. Inclusion criteria were a positive HIV test and age > 18 years. The procedures for recruitment and data collection have been described in detail elsewhere [13]. Inclusion and baseline examinations took place from March 2015 to December 2016 where 1099 participants were included. Of these, 949 participants participated in the 2-year-follow-up examination which took place from April 2017 to April 2019. In the present study, we included participants at the 2-year-follow-up examinations with available fasting serum insulin and plasma glucose, current treatment with ART and HIV RNA < 50 copies/mL, and absence of co-infection with hepatitis B and C. In total 643 participants were included in the present study.

Hepatitis B virus co-infection was defined as positive hepatitis B virus surface antigen. Hepatitis C virus co-infection was defined as positive hepatitis C virus RNA.

Ethical approval was obtained by the Regional Ethics Committee of Copenhagen (H-8-2014-004). Written informed consent was obtained from all participants.

At the 2-year-follow-up, a physical exam including anthropometrics and blood pressure was performed by trained clinical staff according to epidemiologic research standards [14, 15]. Questionnaires were used to collect information regarding smoking, origin, and antihypertensive treatment. Fasting blood samples were collected and serum insulin, plasma glucose, low-density lipoprotein cholesterol (LDL-C), high-density lipoprotein cholesterol (HDL-C), and total cholesterol were measured. Fasting blood samples were collected after ≥ 8 h of fasting. Data regarding HIV infection were obtained from a review of medical charts. Exposure to older generation ART was defined as ever use of thymidine analogues (zidovudine and stavudine) and/or didanosine. Duration of ART was defined as time since initiation of treatment with ART. Well-treated was defined as current treatment with ART and HIV RNA < 50 copies/mL.

### Definition of clinical outcomes

In accordance with the original homeostasis model assessment (HOMA), insulin resistance was calculated using the equation: fasting plasma glucose (mmol/L) × fasting serum insulin (mU/L)/22.5 [16]. Due to the lack of standardized cut-off value for insulin resistance [17], we defined high insulin resistance (high HOMA-IR) as the upper quartile of the HOMA insulin resistance index.

According to WHO guidelines, abdominal obesity was defined as waist-to-hip ratio (WHR) ≥ 0.9 for men and ≥ 0.85 for women and BMI was classified as < 18.5 underweight, 18.5–24.99 normal weight, 25–29.99 overweight, and ≥ 30 kg/m^2^ obese [18]. According to the questionnaires smoking status was defined as either current smoker, previous smoker or never smoker.

### Statistics

We reported frequency counts and percentages for categorical data and continuous data with means and standard deviations for normal deviates and medians with interquartile ranges (IQR) for variables not normally distributed.

We investigated the association between high HOMA-IR and traditional and HIV-specific risk factors using a logistic regression model adjusted for age, sex, BMI category, smoking status (previous/current/never smoker), and origin. Traditional risk factors were included in the adjusted model. Additionally, we tested the association between abdominal obesity and insulin resistance by adding abdominal obesity to the predefined model. Furthermore, HIV-specific risk factors (Current CD4^+^ count per 100 cells/µL, CD4^+^/CD8^+^-ratio, CD4^+^ nadir < 200 cells/µL, prior exposure to older generation ART, duration of treatment with ART and previous AIDS-defining conditions) were investigated by adding them to the model one at a time. When including exposure to older generation ART and duration of ART in the model, we also adjusted for time with HIV.

A possible effect modification by prior exposure to older generation ART on the association between significant risk factors and high HOMA-IR was explored by adding an interaction term to the model.

In an exploratory analysis using our predefined model we investigated the association between high HOMA-IR and hip measurements and waist measurements tested separately. Additionally, we tested the association between time with HIV and high HOMA-IR using our predefined model.

A P-value of < 0.05 was considered statistically significant. All P-values were two-sided.

## Results

Among the included participants, the median (IQR) age was 52 years (46–61), 87% (n = 557) were male, and 52% (n = 337) had prior exposure to older generation ART, Table 1. Median [IQR] index of IR was 1.86 [1.23–3.14] mmol/L × mU/L.

**Table 1:** Clinical and demographic characteristics of the study population

Variable	PLWH (n = 643)
Age, years, median (IQR)	52 (46, 61)
Sex (male), n (%)	557 (87)
Scandinavian or other European origin, n (%)	562 (87)
BMI (kg/m^2^), median (IQR)	25 (23, 27)
Underweight, BMI < 18.5, n (%)	13 (2)
Normal weight, BMI 18.5-24.99, n (%)	319 (50)
Overweight, BMI25-29.99, n (%)	234 (36)
Obese, BMI ≥ 30, n (%)	77 (12)
Abdominal obesity, n (%)	372 (58)
Smoking status	
Current, n (%)	154 (24)
Previous, n (%)	218 (34)
Never, n (%)	266 (41)
Hypertension, yes, n (%)	317 (49)
Transmission mode	
MSM, n (%)	461 (72)
Heterosexual, n (%)	140 (22)
IDU, n (%)	5 (0.8)
Other, n (%)	10 (1.6)
Current CD4^+^, cells/µL, median (IQR)	660 (515, 831)
< 200	10 (1.6)
200–349	25 (4)
350–499	101 (16)
≥ 500	467 (73)
CD4^+^ nadir < 200, cells/µL, n (%)	252 (39)
CD4^+^/CD8^+^ ratio, median (IQR)	0.9 (0.6, 1.2)
Time since HIV diagnosis, years, median (IQR)	16 (9, 24)
History of AIDS (yes), n (%)	111 (17)
Time with ART treatment, years, median (IQR)	13 (7, 20)
Exposure to older generation ART, yes, n (%)	337 (52)

Hypertension was defined as systolic blood pressure ≥ 140 mmHg and/or diastolic blood pressure ≥ 90 mmHg and/or antihypertensive treatment

MSM: men who have sex with men; IDU: injecting drug use; ART: combined antiretroviral therapy

### Traditional risk factors associated with high HOMA-IR

**Fig. 1:**
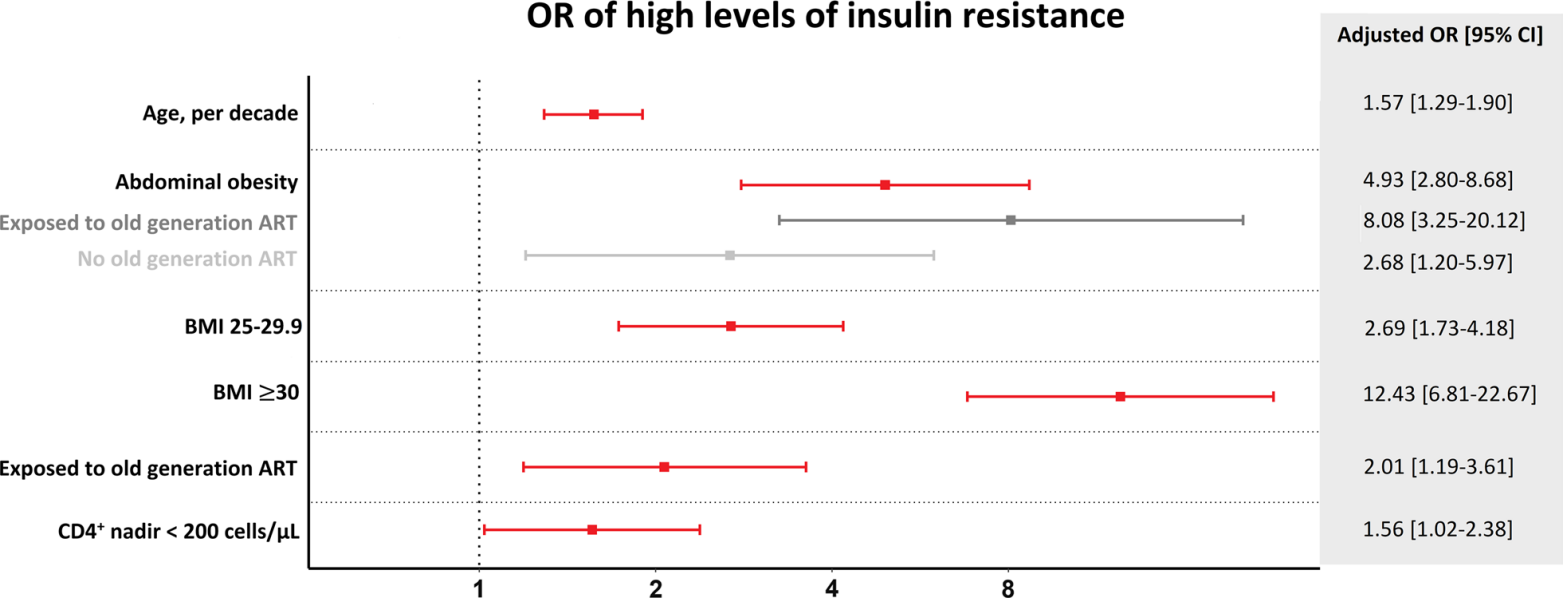
Adjusted OR calculated using a logistic regression model adjusted for age, sex, BMI, smoking status, and origin. When including exposure to older generation ART in the model we also adjusted for time with HIV

Traditional risk factors significantly associated with high HOMA-IR included age (adjusted OR [aOR] = 1.57, per decade older, [95% CI 1.29–1.90], *P* < 0.001), BMI 25–29.9 (aOR = 2.69 [1.73–4.18], compared to BMI 20–24.9, *P* < 0.001), BMI ≥ 30 (aOR = 12.43 [6.81–22.67], compared to BMI 20–24.9, *P* < 0.001) and abdominal obesity (aOR = 4.93 [2.80–8.68], *P* < 0.001), Fig. 1. Other traditional risk factors such as smoking status, origin, and sex were not associated with high HOMA-IR, Table 2. Furthermore, we found waist measurements to be associated with high HOMA-IR (aOR = 1.11, per cm, [1.07;1.14], *P* < 0.001), while hip measurements were not (aOR = 1.01, per cm, [0.97;1.04], *P* = 0.768).

**Table 2:** Risk factors associated with high HOMA-IR

Traditional risk factors	OR [95% CI]	*P*-value	aOR [95% CI]	*P*-value
Age, per 10 years older	1.45 [1.22–1.69]	**< 0.001**	1.57 [1.29–1.90]	**< 0.001**
Male sex	0.86 [0.52–1.43]	0.556	1.04 [0.57–1.90]	0.894
Abdominal obesity	7.34 [4.49–12.00]	**< 0.001**	4.93 [2.80–8.68]	**< 0.001**
Waist circumference, per cm	1.10 [1.08–1.13]	** < 0.001**	1.11 [1.07–1.14]	**< 0.001**
Hip circumference, per cm	1.08 [1.05–1.10]	** < 0.001**	1.01 [0.97–1.04]	0.768
BMI				
Normal weight, BMI 18.5-24.99	Ref		Ref	
Overweight, BMI 25-29.99	2.56 [1.67–3.91]	**< 0.001**	2.69 [1.73–4.18]	**< 0.001**
Obese, BMI ≥ 30	10.53 [6.06–18.31]	**< 0.001**	12.43 [6.81–22.67]	**< 0.001**
Smoking				
Never smoker	Ref		Ref	
Current smoker	0.84 [0.52–1.34]	0.453	1.29 [0.76–2.19]	0.347
Previous smoking	1.19 [0.79–1.78]	0.405	1.10 [0.69–1.74]	0.686
Non-European origin	1.16 [0.67–2.01]	0.598	1.25 [0.65–2.40]	0.497
HIV-specific risk factors				
Exposure to older generation ART*	2.35 [1.62–3.41]	**< 0.001**	2.14 [1.24–3.71]	**0.006**
Duration of ART, per 5 years*	1.38 [1.20–1.59]	**< 0.001**	1.33 (0.99–1.79)	0.054
Time with HIV, per 5 years	1.26 [1.14–1.39]	**< 0.001**	1.19 [1.04–1.35]	**0.009**
CD4^+^ count, per 100 cells/µL	1.00 [1.00–1.00]	0.285	1.00 [1.00–1.00]	0.333
CD4^+^ nadir < 200 cells/µL	1.69 [1.17–2.43]	**0.005**	1.56 [1.02–2.38]	**0.038**
CD4^+^/CD8^+^-ratio, per 0.1 change in ratio	0.79 [0.53–1.17]	0.237	0.81 [0.53–1.24]	0.326
Previous AIDS defining condition	1.61 [1.04–2.51]	**0.033**	1.39 [CI 0.83–2.30]	0.209

Adjusted OR calculated using logistic regression in a model adjusted for age, sex, BMI, smoking status, and origin

^*^Calculated using our adjusted model, further adjusting for time with HIV

### HIV-specific risk factors associated with high HOMA-IR

Exposure to older generation ART (aOR = 2.14 [1.24–3.71], *P* = 0.006), and nadir CD4^+^ < 200 cells/µL (aOR = 1.56 [1.02–2.38], *P* = 0.038) were significantly associated with high HOMA-IR, Fig. 1. Current CD4^+^ count per 100 cells/µL, CD4^+^/CD8^+^-ratio, duration of ART and previous AIDS defining condition were not associated with high HOMA-IR, Table 2. In exploratory analysis we found time with HIV to be associated with high HOMA-IR (aOR = 1.19, per 5 years, [1.04–1.35], *P* = 0.009).

### Effect modification by prior exposure to older generation ART

When investigating effect modification by prior exposure to older generation ART, we found the association between abdominal obesity and high HOMA-IR to be stronger in PLWH with prior exposure to older generation ART (aOR = 8.08 [3.25–20.12] vs aOR = 2.68 [1.20–5.97], *P*-interaction = 0.042), Fig. 1. We found no effect modification between older generation ART and other risk factors.

## Discussion

In this study of well-treated PLWH, abdominal obesity, BMI ≥ 25, exposure to older generation ART, and CD4^+^ nadir < 200 cells/µL was associated with insulin resistance. Furthermore, the association between abdominal obesity and insulin resistance was stronger in PLWH with previous use of older generation ART. This suggests that insulin resistance in PLWH is related to both exposure to older generation ART and prior immunodeficiency.

Insulin resistance is a key determinant in the development of T2DM [19]. In the COCOMO study, we have previously reported an 1.7 increased odds of T2DM in PLWH compared to uninfected individuals [4]. This finding is consistent with findings from other countries [2, 3]. In this study, among the traditional risk factors, age, BMI ≥ 25 and abdominal obesity was associated with insulin resistance in PLWH. These are all well-described risk factors for both insulin resistance and T2DM [6]. Especially, abdominal obesity, including increased amounts of visceral fat, is thought to be associated with a high risk of developing insulin resistance [7, 8].

Among HIV-specific risk factors, exposure to older generation ART was associated with insulin resistance, even when adjusting for time with HIV. This is consistent with other studies associating exposure to older generation ART and T2DM [9, 10]. In muscle and adipose tissue older generation ART has been shown to inhibit glucose transporter 4 (GLUT4) [5, 20, 21] that contributes to glucose homeostasis. Accordingly, inhibition of GLUT4 may lead to insulin resistance which may explain the association between older generation ART and insulin resistance. Furthermore, older generation ART has been associated with changes in adipose tissue including lipodystrophy [11, 12]. Interestingly, the association between abdominal obesity and insulin resistance was stronger in PLWH with prior exposure to older generation ART. This may suggest that the alterations of adipose tissue in PLWH exposed to older generation ART increase the effect of abdominal obesity on insulin resistance.

In addition to older generation ART, CD4^+^ nadir < 200 cells/µL was associated with insulin resistance. This finding is consistent with other studies reporting lower CD4^+^ nadir to be associated with an increased risk of T2DM [2, 22]. Furthermore, both insulin resistance and prior immunodeficiency have been associated with cardiovascular comorbidity in PLWH [23].

There were limitations to this study. First, the study is cross-sectional which prevents us from drawing any conclusions regarding causality. Second, since there is no standardized cut-off value for insulin resistance the clinically significant insulin resistance is unknown. Third, we could not compare insulin resistance in PLWH to insulin resistance in uninfected controls because we did not have access to a control population with fasting blood samples. The strengths of the study include the large cohort of well-treated PLWH and the use of fasting blood samples to define insulin resistance.

## Conclusions

In conclusion, in well-treated PLWH with absence of chronic hepatitis B or C infection, insulin resistance was associated with abdominal obesity, exposure to older generation ART, and CD4^+^ nadir < 200 cells/µL. This may suggest that insulin resistance in PLWH is associated with both prior immunodeficiency and metabolic changes due to exposure to older generation ART. Furthermore, the association between abdominal obesity and insulin resistance was stronger in PLWH with exposure to older generation ART than in PLWH without exposure to these drugs, suggesting older generation ART to have long-lasting effects on abdominal adipose tissue related to insulin resistance. This suggests that special attention on blood glucose in these patients could be beneficial.

## Data Availability

The datasets generated during and/or analysed during the current study are available from the corresponding author on reasonable request. Danish legislation does not allow the dataset to be freely available.

## Abbreviations

aOR: Adjusted OR
ART: Antiretroviral therapy
COCOMO Study: Copenhagen Comorbidity in HIV Infection Study
GLUT4: Glucose transporter 4
HDL-C: High-density lipoprotein cholesterol
HOMA: Homeostasis model assessment
IQR: Interquartile range
LDL-C: Low-density lipoprotein cholesterol
PLWH: People living with HIV
T2DM: Type 2 diabetes mellitus
